# Relationship between relative fat mass and infertility: A cross-sectional study

**DOI:** 10.1097/MD.0000000000039990

**Published:** 2024-10-11

**Authors:** Xi Zhao, Yue Wu, Huangyu Hu

**Affiliations:** a Department of Integrated Traditional Chinese and Western Medicine, Nanchong Central Hospital, Capital Medical University Affiliated Beijing Anzhen Hospital Nanchong Hospital, The Second Clinical Medical College, North Sichuan Medical College, Nanchong, China; b Pelvic Floor Rehabilitation Center, Sichuan University West China Second University Hospital, Chengdu, China; c Acupuncture School of Hospital of Chengdu University of Traditional Chinese Medicine, Chengdu, China.

**Keywords:** infertility, NHANES, obesity, relative fat mass

## Abstract

Infertility is closely related to obesity. Relative fat mass (RFM) is a newer index for assessing percentage of body fat, which reflects the amount of body fat better than body mass index (BMI), but its relationship with infertility needs further study. The purpose of this study was to assess whether there was an association between RFM and infertility in women aged 20 to 44 years in the United States. The corresponding participants were selected from the National Health and Nutrition Examination Survey (NHANES) 2013 to 2018. RFM was used as the independent variable and infertility as the dependent variable. Multiple logistic regression and generalized additive models were used to explore the association between RFM and infertility, 2-stage linear regression models were used to calculate threshold effects, and subgroup analyses and tests of interactivity were used to find sensitive populations. A total of 2328 women aged 20 to 44 years were included. In the fully adjusted model, the risk of infertility increased by 6% for each increase in RFM (OR = 1.06, 95% CI: 1.00–1.12). There was a nonlinear relationship between RFM and infertility with 2 breakpoints of K1 = 31.04 and K2 = 48.4. There was a positive association between RFM and infertility on the right side of K1 and, on the left side of K2 (OR = 1.08, 95% CI: 1.01–1.16; OR = 1.07, 95% CI: 1.01–1.14). In contrast, no statistically significant association between RFM and infertility was found on the left side of K1 as well as on the right side of K2. There was a nonlinear relationship between RFM and infertility, with a positive association with infertility when RFM was in the range of 31.04 to 48.4.This suggests that RFM may be an alternative to BMI in the management of obese infertile women, but this needs to be further confirmed by prospective studies.

## 1. Introduction

The inability to get pregnant within a year of unprotected sex is known as infertility.^[1]^ Its prevalence varies a little bit from nation to nation, with a reported prevalence of 12.5% in the United Kingdom and 25% in China.^[2,3]^ According to a research from the United States, 6% of women of reproductive age had infertility diagnoses, and 12% had impaired fertility.^[4]^ Women’s mental health is negatively impacted by infertility, with problems like anxiety and sadness among others.^[5]^ Infertility is linked to a number of subsequent chronic illnesses, including cancer, diabetes, and cardiovascular disease, according to research.^[6,7]^ In addition to this, infertility has significant public health implications, including marriage, and economic burden.^[8,9]^

With obesity rates sharply rising over the past few decades, obesity is a major public health concern on a global scale.^[10]^ According to studies, obesity will affect nearly half of all American people by the year 2030.^[11]^ Many chronic diseases are more common as a result of obesity, and it also has an impact on women’s fertility.^[12,13]^ Several studies support obesity’s role as a distinct risk factor for female infertility.^[14,15]^ Although assisted reproductive technologies are frequently used to treat infertility, it’s important to remember that losing weight may still be a good approach to increase your chances of becoming pregnant.^[16]^ Obesity is defined as a complication of excess adipose tissue, and excess adipose tissue contributes significantly to the development of various diseases.^[17]^

Relative fat mass (RFM) is a new indicator that evaluates the percentage of body fat based on the height to waist ratio.^[18]^ Body mass index (BMI) is commonly used to assess obesity, but it does not reflect the true body fat well. It also does not reflect the loss of muscle mass and relative increase in fat mass due to aging.^[19]^ Dual-energy X-ray absorptiometry is a more accurate measure of body composition, however, it is radioactive and has poor utility.^[20]^ RFM has been shown to provides a better estimate of body fat percentage measured by dual-energy X-ray absorptiometry than BMI.^[18]^ RFM is more closely related to type 2 diabetes occurrence than conventional indices like BMI and waist circumference (WC), according to prior research.^[21]^ Although RFM is a marker for assessing whole body fat percentage, there is no prior research linking it to infertility. We looked into the connection between RFM and infertility using information from the National Health and Nutrition Examination Survey (NHANES). We predicted that the prevalence of infertility would be positively correlated with RFM levels.

## 2. Material and methods

### 2.1. Data sources and study population

All data were obtained from the NHANES database, which refers to population-based cross-sectional surveys, and the study was authorized by the United States Ethics Committee and all participants provided informed written consent.

**Figure 1.**
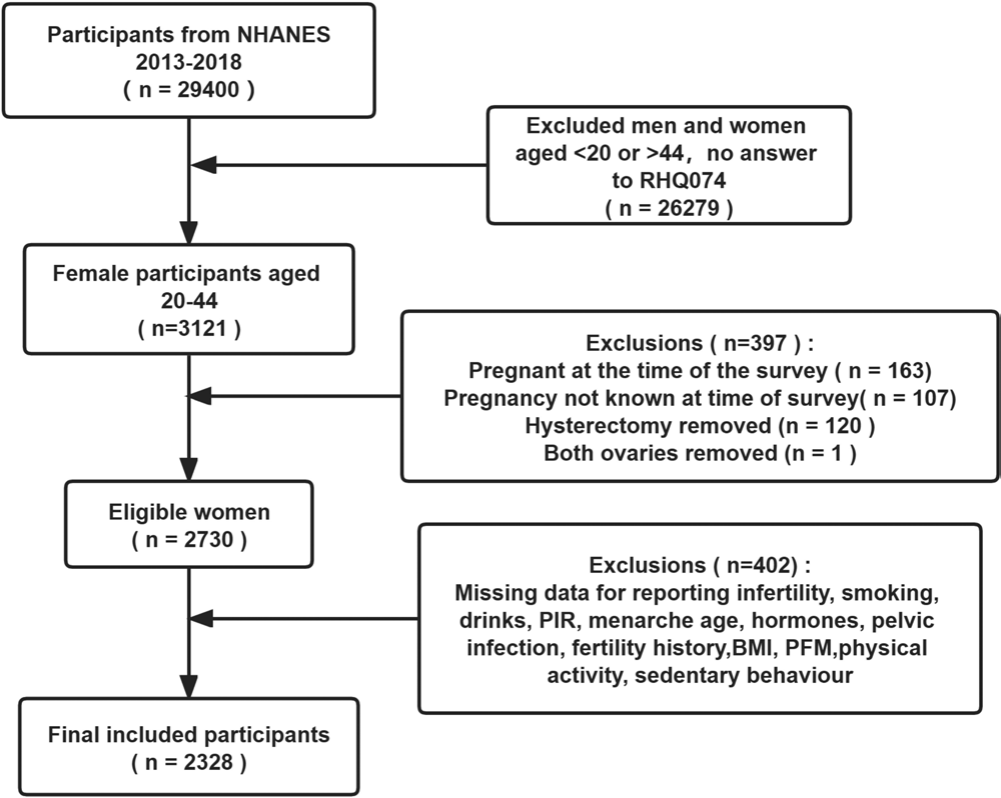
Flowchart of study population selection. BMI = body mass index, NHANES = National Health and Nutrition Examination Survey, PIR = poverty income ratio, RFM = relative fat mass.

We chose NHANES data from 3 consecutive 2-year NHANES cycles (2013–2014, 2015–2016 and 2017–2018). First, women between the ages of 20 and 44 who answered question RHQ074 were chosen (n = 3121). Women who were pregnant at the time of the survey (n = 163) and those who were pregnant but did not know it (n = 107) were then excluded. Women with hysterectomy (n = 120), bilateral oophorectomy (n = 1), missing PFM, smoking, drinks, poverty income ratio (PIR), menarche age, hormones, pelvic infections, BMI, physical activity, sedentary time, and pregnancy history were excluded (n = 402). Finally, 2328 women were included as data for analysis. Figure 1 depicts the data selection procedure.

### 2.2. Dependent variable

Women with infertility were identified based on their responses to RHQ074 in the reproductive health questionnaire section “Have you ever attempted to become pregnant over a period of at least a year without becoming pregnant?” Women who answered “yes” were thought to be infertile, whereas those who answered “no” were thought to be normal.

### 2.3. Independent variable

The height and waist circumference from the NHANES database’s inspection section are used to generate the RFM. RFM is calculated as 64 − (20 × (height/waist)) + (12 × sex), where sex = 0 for men and sex = 1 for women.^[18]^

### 2.4. Covariates

In this study, covariates were used based on previous studies as well as based on clinical practice.^[22–24]^ The covariates used included age, race, education, marital status, PIR, BMI, smoking, drinks, physical activity, sedentary behavior, regular menstruation, menarche age, hormones, pelvic infections, and pregnancy history. These covariates were obtained from the NHANES database of demographics, examinations, physical activity questionnaires from questionnaires, reproductive health questionnaires, smoking questionnaires and alcohol consumption questionnaires.

### 2.5. Statistical analysis

Based on self-reported infertility, the study population was split into 2 groups, and descriptive analyses of the demographic and measurement indicators were carried out. For comparison between groups, categorical data were expressed as frequency (composition ratio) [n (%)], continuous variables were expressed as mean standard deviation (mean ± SD), and continuous variables were tested using Kruskal–Wallis test and categorical variables were tested using chi-square tests. RFM was categorized by tertile (Tertile 1, Tertile 2, Tertile 3). The association between RFM and infertility risk was analyzed by multiple regression models using the Tertile 1 category as a reference, with results expressed as an odds ratio (OR) and 95% confidence interval (95% CI). Specifically, Model 1 did not adjust for any variables. Model 2 was adjusted for age and race. In addition, Model 3 builds on Model 2 for education, martial status, PIR, BMI, physical activity, sedentary behavior, drinks, smoking, menarche age, pelvic infection, regular menstruation, hormones, pregnancy history were adjusted. A generalized additive model and smoothed curve fitting were used to address the nonlinearity of RFM and infertility. A threshold effects analysis model was used to investigate the association and inflection points between RFM and infertility. We then performed subgroup analyses to observe the association between RFM and infertility across populations. Lastly, since the NHANES database is based on a complex sampling design, it is necessary to account for the sample weights. In the sensitivity analysis, we incorporated these sample weights into a weighted regression. We analyzed the data using the Empower software (version 2.0, http://www.empowerstats.com, X&Y Solutions, Inc. Boston, MA, USA) and the R statistical package (version 3.4.3, http://www.r-project.org). The standard for significance was a *P* value of <.05.

## 3. Results

### 3.1. Baseline characteristics

Table 1 shows demographic characteristics and other covariates for a total of 2328 women aged 20 to 44 years who were included in our study for analysis. In this study, 259 of the 2328 participants were infertility, and the prevalence of infertility was 11.12%. The overall mean age of the participants was 32.23 ± 7.25, 34.78 ± 6.58 in the infertility group and 31.91 ± 7.27 in the control group. In the subgroup based on the presence of infertility, the differences were statistically significant for age, marital status, BMI, RFM, smoke, pelvic infections, and pregnancy history. The differences in race, education, PIR, physical activity, sedentary time, drinks, menarche age, regular menstruation, and hormones were not statistically significant. Women who were older, smoked, had a history of pelvic infections, had previous births, had a higher BMI, and had RFM appeared to be more likely to develop infertility than women who were not diagnosed with infertility.

**Table 1:** Baseline characteristics of study population according to infertility.

Variables	Total	Control	Infertility	*P* value
	n = 2328	n = 2069	n = 259	
Age, mean + SD	32.23 ± 7.25	31.91 ± 7.27	34.78 ± 6.58	<.001*
Race, n (%)				.334
Mexican American	385 (16.54%)	346 (16.72%)	39 (15.06%)	
Other Hispanic	230 (9.88%)	212 (10.25%)	18 (6.95%)	
Non-Hispanic White	808 (34.71%)	709 (34.27%)	99 (38.22%)	
Non-Hispanic Black	487 (20.92%)	426 (20.59%)	61 (23.55%)	
Non-Hispanic Asian	295 (12.67%)	267 (12.90%)	28 (10.81%)	
Other race	123 (5.28%)	109 (5.27%)	14 (5.41%)	
Education, n (%)				.740
Under high school	314 (13.49%)	283 (13.68%)	31 (11.97%)	
High school or equivalent	442 (18.99%)	391 (18.90%)	51 (19.69%)	
College graduate or above	1572 (67.53%)	1395 (67.42%)	177 (68.34%)	
Marital status, n (%)				<.001*
Married/living with partner	1326 (56.96%)	1144 (55.29%)	182 (70.27%)	
Widowed/divorced/separated	243 (10.44%)	208 (10.05%)	35 (13.51%)	
Never married	759 (32.60%)	717 (34.65%)	42 (16.22%)	
PIR, mean + SD	2.36 ± 1.61	2.34 ± 1.61	2.53 ± 1.66	.072
BMI, mean + SD	29.52 ± 8.39	29.25 ± 8.34	31.69 ± 8.53	<.001*
RFM, mean + SD	41.03 ± 6.38	40.80 ± 6.38	42.87 ± 6.11	<.001*
Physical activity, mean + SD	230.89 ± 377.58	233.46 ± 382.05	210.39 ± 339.81	.354
Sedentary behavior, mean + SD	374.21 ± 204.98	372.81 ± 203.72	385.37 ± 214.87	.353
Drinks, n (%)				.941
Yes	553 (23.75%)	480 (23.20%)	75 (28.96%)	
No	1775 (76.25%)	1578 (76.27%)	197 (76.06%)	
Smoking, n (%)				.040*
Yes	555 (23.84%)	480 (23.20%)	75 (28.96%)	
No	1773 (76.16%)	1589 (76.80%)	184 (71.04%)	
Menarche age, mean + SD	12.56 ± 1.80	12.58 ± 1.77	12.42 ± 1.97	.423
Pelvic infection, n (%)				<.001*
Yes	110 (4.73%)	87 (4.20%)	23 (8.88%)	
No	2218 (95.27%)	1982 (95.80%)	236 (91.12%)	
Regular menstruation, n (%)				.553
Yes	2173 (93.34%)	1929 (93.23%)	244 (94.21%)	
No	155 (6.66%)	140 (6.77%)	15 (5.79%)	
Hormones, n (%)				.204
Yes	62 (2.66%)	52 (2.51%)	10 (3.86%)	
No	2266 (97.34%)	2017 (97.49%)	249 (96.14%)	
Pregnancy history, n (%)				<.001*
Yes	1641 (70.49%)	1426 (68.92%)	215 (83.01%)	
No	687 (29.51%)	643 (31.08%)	44 (16.99%)	

BMI = body mass index, PIR = ratio of family income to poverty, RFM = relative fat mass.

* *P* < .05.

### 3.2. Higher RFM associated with risk of infertility

We used multiple regression analysis to assess the association between RFM and infertility in 3 different models (Table 2). The 3 models were model 1, model 2 and model 3, where model 1 was not adjusted for covariates and model 2 was adjusted for age and race. Finally, for model 3, we adjusted for age, race, education, marriage, PIR, alcohol consumption, smoking, BMI, physical activity, sedentary activity, menarche age, regular menstruation, pelvic infection, pregnancy history and hormones. As can be seen in Table 2, there was a positive association between RFM and infertility in all 3 models. In the fully adjusted model 3, it was shown that for every 1 unit increase in RFM, the risk of infertility rose by 6% (OR = 1.06, 95% CI: 1.00–1.12). We converted RFM from a continuous variable to a categorical variable (tertile) for sensitivity analysis. Using the lowest tertile as a reference, the risk of infertility increased with increasing RFM. In the fully adjusted model, the ORs were 1.49 for Tertile 2 and 2.49 for Tertile 3 compared to Tertile 1 (Tertile 2: OR = 1.49, 95% CI: 1.00–2.23; Tertile 3: OR = 2.49, 95% CI: 1.41–4.40; *P* for trend = .0019).

**Table 2:** Association between RFM and infertility.

Related fat mass group	Crude model (Model 1)	Minimally adjusted model (Model 2)	Fully adjusted model (Model 3)
RFM/OR (95% CI)			
Continuous	1.05 (1.03, 1.08)	1.04 (1.02, 1.07)	1.06 (1.00, 1.12)
Categories			
Tertile 1 (22.03–38.06)	Reference	Reference	Reference
Tertile 2 (38.07–44.27)	1.57 (1.10, 2.24)	1.46 (1.02, 2.11)	1.49 (1.00, 2.23)
Tertile 3 (44.28–56.67)	2.42 (1.73, 3.39)	2.16 (1.52, 3.08)	2.49 (1.41, 4.40)
*P* for trend	<.0001*	<.0001*	.0019*

BMI = body mass index, PIR = ratio of family income to poverty, RFM = relative fat mass.

Model 1: no covariates were adjusted. Model 2: adjusted for age and race. Model 3: adjusted for age, race, education, martial status, PIR, BMI, physical activity, sedentary behavior, drink, smoke, menarche age, pelvic infection, regular menstruation, hormones, pregnancy history.

* *P* < .05.

**Figure 2.**
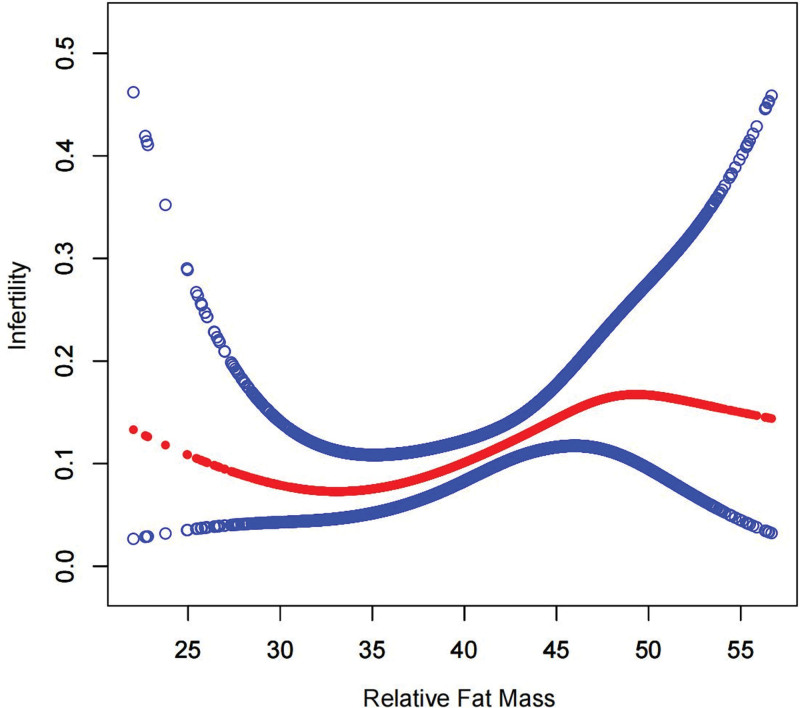
Smooth curve fitting detected a nonlinear positive relationship between RFM and infertility. RFM = relative fat mass.

The nonlinear relationship between RFM and infertility was fitted using a smoothed curve (Fig. 2). We further calculated 2 breakpoints at K1 = 31.04 and K2 = 48.4. On the right side of K1, there was a positive association between RFM and infertility (OR = 1.08, 95% CI: 1.01–1.16). Similarly, on the left side of K2, there was a positive association between RFM and infertility (OR = 1.07, 95% CI: 1.01–1.14). In contrast, no statistically significant association between RFM and infertility was found on the left side of K1 as well as on the right side of K2. The log-likelihood ratio test *P* values were .042 and .025, respectively (Table 3). This result suggests that RFM was positively associated with infertility between 31.04 and 48.4.

**Table 3:** Threshold effect analysis of RFM and infertility using a 2-piecewise linear regression model.

		For breakpoint1		For breakpoint2
Model 1	OR (95% CI)	1.05 (0.98, 1.12)	OR (95% CI)	1.09 (1.02, 1.16)
Model 2	Breakpoint (K1)	31.04	Breakpoint (K2)	48.4
	OR1 (< 31.04)	0.83 (0.67, 1.02)	OR1 (< 47.05)	1.07 (1.01 1.14)
	OR2 (>31.04)	1.08 (1.01, 1.16)	OR2 (> 47.05)	0.89 (0.73, 1.07)
	OR2/OR1	1.30 (1.04, 1.64)	OR2/OR1	0.83 (0.70, 0.98)
	Logarithmic likelihood ratio test *P* value	0.042*	Logarithmic likelihood ratio test *P* value	0.025*

Model 1: Standard linear model. Model 2: Two-piecewise linear model.

RFM = relative fat mass.

* *P* < .05.

### 3.3. RFM is positively associated with increased risk of infertility in different subgroups

**Figure 3.**
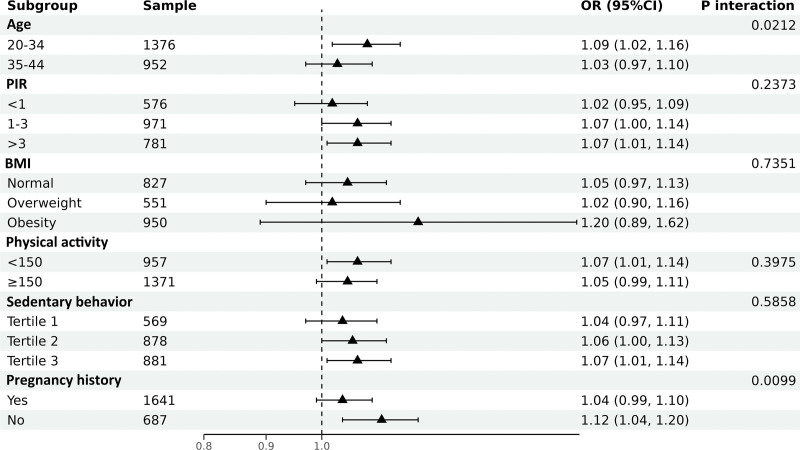
Subgroup analysis for the association between RFM and infertility. RFM = relative fat mass.

We then performed subgroup analyses to assess whether the association between RFM and infertility was consistent across populations. We stratified by age, PIR, BMI, physical activity, sedentary behavior, and pregnancy history, and also performed interaction tests (Fig. 3). The interaction test helps us to determine whether the effect modifiers are significantly dependent on this association. Our results suggest that these associations are not consistent. Age, previous pregnancy or not had a significant interaction (*P* for interaction < .05), while PIR, BMI, physical activity and sedentary behavior were not statistically significant (*P* for interaction > .05). A positive association was observed in all subgroups. In conclusion, the relationship between RFM and infertility was age and pregnancy history dependent, and it may be more applicable to people aged 20 to 34 years with no pregnancy history.

## 4. Sensitivity analysis

We conducted a sensitivity analysis considering sample weights, with detailed results presented in Tables S1, Supplemental Digital Content, http://links.lww.com/MD/N687 and S2, Supplemental Digital Content, http://links.lww.com/MD/N687 In the fully adjusted weighted multivariate regression analysis, the findings were largely consistent with the unweighted results, showing a positive association between RFM and infertility (OR = 1.05, 95% CI: 1.01–1.09). Similarly, the results were also consistent in the subgroup analysis.

## 5. Discussion

This research examined the connection between RFM and infertility in American women between the ages of 20 to 44. In our research, we discovered a correlation between RFM and infertility when it ranged between 31.04 and 48.4. The correlation between RFM and infertility was not statistically significant, however, when RFM was <31.04 or >48.4. Additionally, RFM may be more appropriate in people between the ages of 20 to 34 who have never been pregnant when used to forecast the likelihood of infertility.

This would be the first research, to our knowledge, to look into the relationship between RFM and infertility. To measure the percentage of body fat, RFM can be used as an indicator. Although BMI is the metric that is most frequently used to define obesity, it is not a reliable indicator of body fat percentage. For instance, a BMI over 30 would exclude about 50% of the females whose body fat percentage is considered obese.^[25]^ Since obesity is the result of an excessive buildup of body fat, it is important and helpful to have a metric that accurately depicts the excessive fat status. Previous research has shown that RFM has a lower rate of obesity misclassification than BMI, and that RFM can be used to predict obesity-related type 2 diabetes, and heart failure.^[18,21,26]^ Higher levels of RFM within a certain range were associated with the risk of infertility in our study, and all showed a positive association in the BMI subgroup. Obesity is well known to be strongly linked to fertility. Obesity was linked to lower fertility in a prospective study conducted in the United States, regardless of normal menstrual cycles.^[27]^ Obese women also have a poorer response to infertility treatment than normal women. A meta-analysis of 21 studies clearly demonstrated a negative relationship between female obesity and live birth rates in in vitro fertilization (IVF).^[28]^ In contrast, obesity interventions increased the rate of spontaneous conception in women.^[29,30]^ Levels of adipokines, which are secreted primarily by white adipose tissue, are altered in the obese state.^[31]^ And adipokines are signals between energy metabolism and reproduction.^[32]^ These common adipokines are Leptin, Lipocalin, Chemerin, Visfatin, Resistin and Apelin.^[32]^ For example, obesity can lead to leptin resistance, and this can decrease gonadotropin-releasing hormone (GnRH) secretion, reducing fertility and even infertility.^[33]^ In contrast to leptin, lipocalin is significantly lower in obese people, which affects endometrial tolerance and embryo implantation.^[34]^ Chemerin is expressed in human ovarian granulosa cells, and follicular fluid levels of chemerin are at least 57-fold higher than plasma, and biologically active, locally enhanced chemerin may negatively affect follicular development and oocyte maturation.^[35]^ Visfatin exerts an insulin-mimetic effect by binding to insulin receptors, which in turn results in the development of insulin resistance.^[36]^ In short, adipokines affect female reproduction through multiple pathways.

RFM was also found to have a higher predictive value in women aged 20 to 34 years than in those aged 35to 44 years in our study. Fertility declines with age, and studies have shown that fertility begins to decline around the age of 32 and declines rapidly after the age of 37.^[37]^ Female fertility declines with age because the ovaries are the most sensitive to aging of all the tissues and organs in the female body.^[38]^ The loss of female fertility is determined by the age-dependent decline in the functional reserve of the ovaries within the expected range. Furthermore, the uterus is closely associated with pregnancy. The uterus’ sensitivity to progesterone and endometrial tolerance are affected by age-related uterine aging.^[39]^ Although RFM is associated with infertility in the age subgroup, the effect of age on fertility for infertile women aged 35 to 44 years may be much greater than that of obesity. It has long been recognized that women over 35 years of age have dramatically elevated levels of circulating follicle stimulating hormone(FSH), which is countered by a decline in fertility.^[40]^ At the same time, it has been shown that elevated FSH is associated with increased body fat and percentage of subcutaneous fat.^[41]^ Animal experiments have also demonstrated that FSH and follicle stimulating hormone receptor (FSHR) are targets for the regulation of fat metabolism, and that fat accumulation in normal female mice can be significantly reduced by blocking the interaction between the two.^[42]^ In turn, these excess fat accumulations can cause further damage to fertility and subsequent infertility through the previously mentioned pathways. This shows that obesity is not a major cause of infertility in older women (age > 35).

Furthermore, RFM predicted a higher value in infertile women with no history of pregnancy than in those with a history of pregnancy in our study. Infertility can be classified into primary and secondary infertility based on the presence or absence of a clinical history of pregnancy.^[1]^ Obesity is thought to play a significant role in primary infertility. Another reason is that altered body shape patterns are associated with fertility.^[43]^ There was less thigh fat and more abdominal fat in childbearing women compared to non-childbearing women, which was unrelated to age or BMI.^[44]^ It could imply that RFM is more concerned with changes in total body fat mass than trunk fat mass.

The large sample size and the sound choice of statistical methods increased the reliability of our study. This study does, nevertheless, have certain shortcomings. First of all, the cross-sectional study methodology used in the present investigation showed a link between RFM and infertility within a range, however the causal explanation was not provided. Thus, large sample size prospective study methods are required in the future to determine the two’s causal relationship. Second, there was some bias in the inclusion sample since it was unable to tell whether male partners had reproductive issues due to restrictions on public databases. Thirdly, residual confounding brought on by unmeasured or unidentified variables cannot be totally ruled out. The infertility diagnosis was made based on self-report, therefore there may have been bias in the recall of past events. Finally, limited by public databases, we lack indicators such as FSH, anti-Müllerian hormone (AMH) and other indicators closely related to fertility, which do not allow for further analyses. In future work we will include more relevant biological indicators to explore the relationship between RFM and them.

## 6. Conclusion

Our study, conducted across 3 survey rounds from 2013 to 2018, revealed a significant positive correlation between RFM and infertility within the range of 31.04 to 48.4. Subgroup analysis further suggested that RFM is especially predictive for infertility in women aged 20 to 34 with no prior pregnancies. In conclusion, RFM within this specific range emerges as a potent predictor of infertility prevalence.

## Acknowledgments

The authors are grateful to the National Health and Nutrition Examination Survey for providing the data used in this study.

## Author contributions

**Conceptualization:** Xi Zhao, Huangyu Hu.

**Data curation:** Xi Zhao, Yue Wu.

**Formal analysis:** Xi Zhao, Yue Wu.

**Investigation:** Xi Zhao, Yue Wu.

**Methodology:** Xi Zhao, Yue Wu, Huangyu Hu.

**Software:** Xi Zhao, Yue Wu.

**Supervision:** Huangyu Hu.

**Validation:** Huangyu Hu.

**Writing – original draft:** Xi Zhao, Yue Wu.

**Writing – review & editing:** Huangyu Hu.

## Supplementary Material


