# Supplementary material for: Relationship between relative fat mass and infertility: A cross-sectional study
Source: Medicine (Baltimore). 2024 Oct 11;103(41):e39990. doi: 10.1097/MD.0000000000039990 (PMC11479528; doi:10.1097/MD.0000000000039990)
Supplement: Supplementary file 1 [file medi-103-e39990-s001.docx]

**Table S1. Association between RFM and infertility,** **weighted**

| **Related fat mass group** | **Crude model**  **(Model 1)** | **Minimally adjusted model (Model 2)** | **Fully adjusted model (Model 3)** |
| --- | --- | --- | --- |
| RFM/OR(95% CI) |  |  |  |
| Continuous | 1.06(1.03,1.10) | 1.05(1.02,1.09) | 1.05(1.01,1.09) |
| Categories |  |  |  |
| Tertile 1(22.03 - 38.06) | Reference | Reference | Reference |
| Tertile 2(38.07 - 44.27) | 1.74(1.11,2.74) | 1.57(0.98,2.53) | 1.43(0.85,2.40) |
| Tertile 3(44.28 - 56.67) | 2.71(1.68,4.37) | 2.40(1.43,4.03) | 2.27(1.31,3.93) |
| P for trend | <0.001* | 0.002* | 0.005* |
| **Notes:** Model 1: no covariates were adjusted. Model 2: adjusted for age and race. Model 3: adjusted for age, race, education, martial status, PIR, BMI, physical activity, sedentary behavior, drink, smoking, menarche age, pelvic infection, regular menstruation, hormones, pregnancy history.*P＜0.05.  **Abbreviations:** RFM, relative fat mass. | | | |

**Table S2. Subgroup analysis for the association between RFM and infertility,** **weighted**

| **Subgroup** | **OR(95%CI)** | **P value** | **P interaction** |
| --- | --- | --- | --- |
| **Age** |  |  | 0.003* |
| 20-34 | 1.08(1.05,1.12) | <0.0001* |  |
| 35-44 | 1.03(0.99,1.07) | 0.13 |  |
| **PIR** |  |  | 0.16 |
| ＜1 | 1.02(0.97,1.07) | 0.42 |  |
| 1-3 | 1.08(1.02,1.14) | 0.01* |  |
| ＞3 | 1.08(1.04,1.12) | <0.001* |  |
| **BMI** |  |  | 0.2 |
| Normal | 1.02(0.97,1.07) | 0.42 |  |
| Overweight | 1.02(0.90,1.16) | 0.78 |  |
| Obesity | 1.03(0.97,1.10) | 0.35 |  |
| **Physical activity** |  |  | 0.14 |
| ＜150 | 1.07(1.04,1.11) | <0.0001* |  |
| ≥150 | 1.04(0.99,1.09) | 0.16 |  |
| **Sedentary behavior** |  |  | 0.47 |
| Tertile 1 | 1.04(1.00,1.09) | 0.03* |  |
| Tertile 2 | 1.07(1.02,1.13) | 0.004* |  |
| Tertile 3 | 1.08(1.03,1.13) | 0.003* |  |
| **Pregnancy history** |  |  | 0.001* |
| Yes | 1.03(1.00,1.07) | 0.04* |  |
| No | 1.14(1.08,1.21) | <0.0001* |  |
| **Notes:***P＜0.05.  **Abbreviations:** RFM, relative fat mass. | | | |
